# Expression Analysis of Cell Wall-Related Genes in the Plant Pathogenic Fungus *Drechslera teres*

**DOI:** 10.3390/genes11030300

**Published:** 2020-03-12

**Authors:** Aurélie Backes, Jean-Francois Hausman, Jenny Renaut, Essaid Ait Barka, Cédric Jacquard, Gea Guerriero

**Affiliations:** 1 Unité de Recherche Résistance Induite et Bio-protection des Plantes—EA 4707, Université de Reims Champagne-Ardenne, UFR Sciences Exactes et Naturelles, SFR Condorcet FR CNRS 3417, Moulin de la Housse—Bâtiment 18, BP 1039, 51687 Reims Cedex 2, France; aurelie.backes@univ-reims.fr (A.B.); ea.barka@univ-reims.fr (E.A.B.); 2 Environmental Research and Innovation (ERIN) Department, Luxembourg Institute of Science and Technology (LIST), L-4940 Hautcharage, Luxembourg; jean-francois.hausman@list.lu (J.-F.H.); jenny.renaut@list.lu (J.R.)

**Keywords:** *Drechslera teres*, barley net blotch, cell-wall-related genes, gene expression

## Abstract

*Drechslera teres* (*D. teres*) is an ascomycete, responsible for net blotch, the most serious barley disease causing an important economic impact. The cell wall is a crucial structure for the growth and development of fungi. Thus, understanding cell wall structure, composition and biosynthesis can help in designing new strategies for pest management. Despite the severity and economic impact of net blotch, this is the first study analyzing the cell wall-related genes in *D. teres*. We have identified key genes involved in the synthesis/remodeling of cell wall polysaccharides, namely chitin, β-(1,3)-glucan and mixed-linkage glucan synthases, as well as endo/exoglucanases and a mitogen-activated protein kinase. We have also analyzed the differential expression of these genes in *D. teres* spores and in the mycelium after cultivation on different media, as well as in the presence of *Paraburkholderia phytofirmans* strain PsJN, a plant growth-promoting bacterium (PGPB). The targeted gene expression analysis shows higher gene expression in the spores and in the mycelium with the application of PGPB. Besides analyzing key cell-wall-related genes, this study also identifies the most suitable reference genes to normalize qPCR results in *D. teres*, thus serving as a basis for future molecular studies on this ascomycete.

## 1. Introduction

Barley is the fourth most-produced cereal in the world behind maize, wheat and rice. This crop is used mostly for animal feed (55%–60%) and by the malt industry (up to 35%) [1]. Globally, twenty million tons of malt are produced per year, and barley is intended for beer production in breweries (https://www.planetoscope.com). However, the production of this monocotyledon may be compromised by the phytopathogen ascomycete *Drechslera teres* [2,3]. This filamentous fungus is responsible for net blotch, easily recognizable by the occurrence of brown necrotic lesions on leaves [4,5]. This disease negatively impacts barley physiology and development and thus causes important agronomic and economic losses. Because of the use of chemical products to control *D. teres*, the emergence of fungicide resistance is a matter of growing concern.

The cell wall is a crucial structure for fungal development, constituting the first physical barrier of protection and is, therefore, a target for antifungal agents [6]. Although the fungal cell wall composition varies from one species to another [6,7], it includes the fundamental components chitin, glucans, mannans and/or galactomannans and glycoproteins [8].

Chitin is one of the main structural components of the fungal cell wall. More specifically, this β-(1,4)-linked homopolymer of *N*-acetylglucosamine (GlcNAc) is synthesized by chitin synthases, CHSs. Chitin plays a crucial role in fungal morphogenesis and in hyphal tip growth [9]. In addition to chitin, several glucans have been identified in fungal cell walls, including β-(1,3)-glucans, mixed-linkage glucans, β-(1,6)-glucans and α-(1,3)-glucans [10].

The *FksA* gene encodes a glycosyltransferase responsible for the synthesis of β-(1,3)-glucans. The inhibition of this gene disrupts the structural integrity of the cell wall and, therefore, impairs fungal development [6]. The majority of mannans and galactomannans decorate cell wall proteins, which are essential for cell wall formation [10]. Multiple classes of fungal cell wall proteins are present with diverse functions: glycosylphosphatidylinositol (GPI)-anchored proteins, transglycosidases, hydrolases including endo/exo-β-(1,3)-glucanase, chitinase, adhesins and hydrophobins. The roles of these proteins are to participate in cytokinesis, to remodel the cell wall and to ensure the adhesion to host cells and tissues [11].

In light of the crucial role played by the fungal cell walls as targets in the development of effective drugs to inhibit pathogens’ growth, we here mine the genome of *D. teres* to identify key genes involved in cell wall biosynthesis [12,13]. The goal is to analyze their expression pattern in the spores and the mycelium grown on different media and in the presence/absence of the plant growth-promoting bacterium (PGPB) *Paraburkholderia phytofirmans*, strain PsJN [14,15,16]. The co-cultivation with a PGPB was investigated because of the increasing attention that biocontrol methods are playing as environmentally friendly strategies to protect crops against pests.

According to many studies, PsJN is able to induce plant growth with an antagonistic effect on pathogens’ development. For instance, the bacterium restricts the spread of the gray mold disease caused by *Botrytis cinerea* [17,18]. PsJN-colonized tomato plants show an improved resistance against *Verticillium* sp. [19].

The remodeling of the cell wall has a crucial impact on the resistance of fungi to drugs [20]. Consequently, deciphering the regulation of cell wall-related genes in *D. teres* could inspire strategies aimed at controlling the spread of the disease. The present results constitute a resource for future molecular studies on the important phytopathogen *D. teres*. Despite the economic impact of the disease caused by *D. teres*, this is, to the best of our knowledge, the first study focusing on the cell wall-related genes of this pathogenic fungus.

## 2. Materials and Methods 

### 2.1. Media and Cultivation Conditions

*D. teres* HE 019 (SAS BAYER Crop Lyon), the asexual form of the ascomycete fungus, was grown on different media, as hereafter described. Growing on these different media, *D. teres* presents several pathotypes. The abbreviations “+”, “−“ and “+/−“ are used to denote rich (+), middle (+/−) and poor (−) media for the whole paper.

#### 2.1.1. Malt Extract Agar (MP)

MP (−), a poor medium, is composed of 10 g/L bacto malt extract and 15 g/L agar. A volume of 150 µL of spores suspension of *D. teres* at a concentration of 50,000 spores/mL is spread on MP (−) medium. After 15 days at 20 °C, *D. teres* produces a small feather duster of approximately 1 cm of height and white in color on this medium. At this stage of development, a transfer from MP (−) media onto BOA (+) media is carried out to maintain *D. teres* and its pathogenicity.

#### 2.1.2. Barley Oat Meal Agar (BOA)

The medium composition is as follows: 18 g/L meal agar, 50 g/L milled leaves of barley and 17 g/L agar. This enriched medium brings all nutritive elements to the growth and spore production of *D. teres*. The fungus was grown for 15 days on this medium with 12 h in darkness and 12 h under blue light near UV emission at 20 °C. Onesirosan and Banttari (1969) demonstrated that spore production was greatest when the fungal cultures were exposed to this wavelength [21]. Two inoculation conditions were tested including the mycelium of the fungus containing spores (first condition) or spore suspension (second condition). For the first type of inoculation, sterile water was deposited on the surface of the fungal mycelium to facilitate its harvest with a rod. For the second inoculation condition, mycelium and spores were filtered through sterile gauze tissues. The filtrate consisted mainly of spores. The concentration of the suspension was adjusted at 10^5^ spores/mL using a Malassez counting chamber (Marienfeld, Lauda-Königshofen, Germany).

#### 2.1.3. Potato Dextrose Agar (PDA)

Co-culture of the fungus and PsJN was carried out on PDA (+/−) medium (39 g/L PDA, pH 4.5 ± 0.2), a medium allowing both the development of fungi and bacteria. After 15 days of growth, a rod was passed on the whole mycelium surface together with sterile water.

### 2.2. Bioinformatics

The maximum likelihood phylogenetic analysis of CHS (protein sequences) was obtained with several CHSs from the following fungi: *D. teres*, *Aspergillus nidulans* [22], *Alternaria alternata*, *Botrytis cinerea* [23], *Blumeria graminis* [24], *Fusarium graminearum*, *Tuber melanosporum* [25] and *Magnaporthe grisea* [9]. The tree was generated with PhyML [26] and available at http://www.phylogeny.fr. The tree was visualized with the online software iTOL (http://itol.embl.de). The CHS sequences from *D. teres, A. alternata*, *B. graminis*, *F. graminearum*, *T. melanosporum* and *M. grisea* were obtained by blasting the *A. nidulans* CHSs (National Center for Biotechnology Information (NCBI) [27]. The identification of CHS domains was carried out with Motif Scan [28]. Sequences were aligned with Clustal Omega [29]. The prediction of the transmembrane domain was performed using the online programs TMHMM (v. 2.0) [30] and Phobius [31]. Protein identifications and corresponding accession numbers from NCBI are indicated in Table 1.

### 2.3. RNA Extraction and cDNA Synthesis

*D. teres* mycelium was crushed with liquid nitrogen using a mortar and a pestle. Spore suspensions, stored at −80 °C, were lyophilized (Freeze Dryer Alpha 1/ 2-4 Christ) for 12 h at −55 °C, then milled using a ball mill MM400 (Retsch) for 2 min at 20 Hz. The extraction of total RNA was carried out using the RNeasy Plant Mini Kit including the DNase I on-column digestion (Qiagen, Leusden, The Netherlands). The integrity of the obtained RNA was evaluated with an Agilent Bioanalyzer (Agilent, Santa Clara, CA, USA). RNA Integrity Numbers (RINs) were >7. The RNA quality and quantity were checked using a Nanodrop ND-1000 spectrophotometer (Thermo Scientific, Villebon-sur-Yvette, France) (A 260/280 and A 260/230 ratios between 1.9 and 2.2). In the case of contamination (ratio 260/230 < 2), samples were precipitated with ammonium acetate (NH_4_OAc) and washed in ethanol [32]. Subsequently, 1 µg of extracted RNA was retro-transcribed using the Superscript II cDNA Synthesis kit (Invitrogen, Carlsbad, CA, USA), according to the manufacturer’s instructions.

### 2.4. Selection and Primer Design of Reference and Target Genes

Eleven putative genes were chosen from the available literature as candidate reference genes [33,34,35]. These genes are actin (EFQ93811.1), aminopeptidase C *ApsC* (EFQ89971.1), cytochrome C oxidase *Cos4* (EFQ89754.1), glyceraldehyde-3-phosphate dehydrogenase *GAPDH* (EFQ89753.1), glucokinase *GlkA* (EFQ96576.1), phosphofructokinase *PfkA* (EFQ89228.1), phosphoglucose isomerase *PgiA* (EFQ88542.1), secretion-associated GTP-binding protein *SarA* (EFQ89474.1), isocitrate dehydrogenase precursor *IsdA* (EFQ94718.1), histone *H2B* (EFQ87126.1) and ribosomal protein S14 *RS14* (EFQ94228.1).

Thirteen target genes were chosen: mitogen-activated-protein kinase *PTK1* (AF272831.1) [36], six isoforms of *CHS*s, i.e., *CHS1, CHS2, CHS3, CHS4, CHS5, CHS7* (EFQ95838.1, EFQ92549.1, EFQ88914.1, EFQ93986.1, EFQ92060.1, EFQ96223.1, respectively), β-(1,3)-glucan synthase *FksA* (EFQ90969.1), endo-β-(1-3)-glucanase *EngA* (EFQ96294.1), three isoforms of putative exo-β-(1,3)-glucanases *ExgB*, *ExgC* and *ExgD* (respectively, EFQ93528.1, EFQ89593.1, EFQ92573.1) and the mixed-linkage glucan synthase *celA* (EFQ94510.1) [22]. All sequences, except *PTK1*, were retrieved by blasting the *A. nidulans* corresponding genes. Primers for qPCR amplification were designed with “Primer3Plus” (http://www.bioinformatics.nl/cgi-bin/primer3plus/primer 3plus.cgi) and analyzed using the OligoAnalyzer tool from Integrated DNA Technologies (http://eu.idtdna.com/calc/analyzer). Primer efficiencies and specificities were calculated and checked using serial dilutions of cDNA with a factor 5 (10, 2, 0.4, 0.08, 0.016, 0.0032 ng/µL). The characteristics of the primers of both reference and target genes are reported in Table 2.

2.5. qPCR: Analysis of the Results and Statistics

A liquid handling robot (epMotion 5073, Eppendorf, Hamburg, Germany) was used to dispense the reaction mixture and cDNA in 384-well plates. The Takyon Low ROX SYBR MasterMix dTTP Blue Kit (Eurogentec, Liège, Belgium) was used for qPCR. The reactions were run in technical triplicates and repeated on four independent biological replicates. PCR was performed on a ViiA 7 Real PCR System (Thermo Scientific, Villebon-sur-Yvette, France) using the following conditions: initial denaturation at 95 °C for 10 min, 40 cycles of denaturation at 95 °C for 15 s, primer annealing at 60 °C for 60 s. At the end of the experiment, the specificity of the amplified products was checked by the analysis of the melting curve. qBase^PLUS^ (version 3.2, Biogazelle, Ghent, Belgium) was used to calculate gene expression. *Cos4* and *PgiA* were identified as the most stable genes in the experimental set-up chosen and as sufficient for normalization. A one-way ANOVA with Tukey’s post-hoc test was performed with IBM SPSS Statistics v19 (IBM SPSS, Chicago, IL, USA) to determine the statistically significant differences among groups.

A hierarchical clustering using uncentered absolute correlation and complete linkage was performed with Cluster 3.0 (Pearson correlation coefficient threshold = 0.83) [37]. The heat map was visualized with Java TreeView (http://jtreeview.sourceforge.net/) [38].

## 3. Results and Discussion

### 3.1. Analysis of D. teres Phenotypes on Several Media

**Figure 1:**
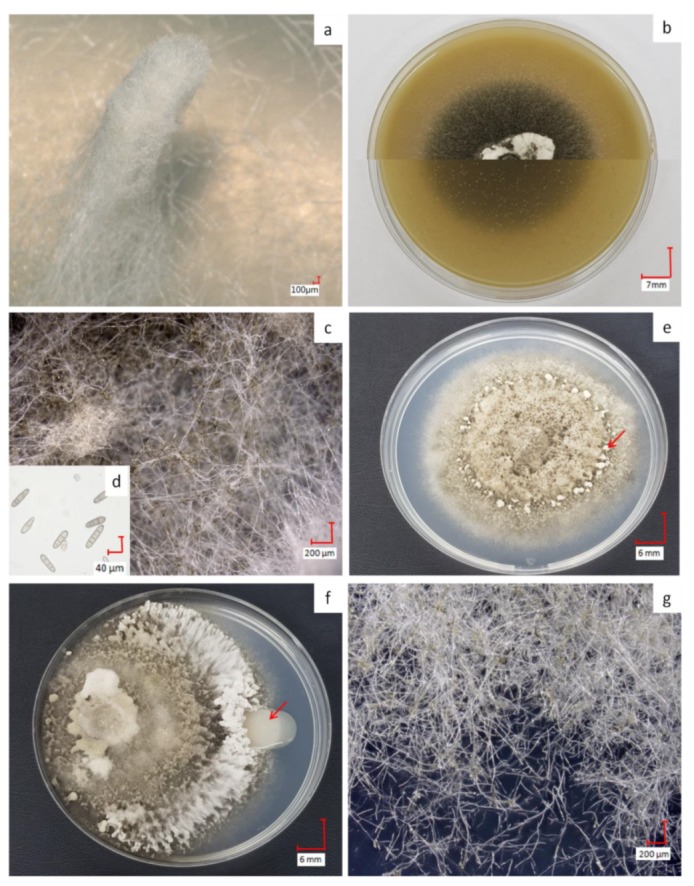
Phenotypes of *D. teres* on malt extract agar (MP) (−) with the typical feather duster (**a**); aspect of *D. teres* on BOA (+) with top and bottom views of the Petri dish in the two halves of the image (**b**); mycelium of *D. teres* on BOA (+) (**c**); transversely septate conidia produced by *D. teres* on BOA (+) (**d**) and *D. teres* phenotype on PDA (+/−) medium with fruiting bodies (red arrow in **e**), aspect in the presence of PsJN (**f**) and mycelium on PDA (+/−) (**g**). The co-culture condition shows that PsJN (red arrow in f) has no visible effect on the mycelium’s growth on PDA (+/−) medium. The pictures (**a,c,d,g**) were obtained with a 3D Keyence VHX-2000F microscope.

*D. teres* shows varying phenotypes according to the culture media used (Figure 1). On MP (−) medium, the fungus develops a white structure, similar to a feather duster (Figure 1a) as described in the literature [39]. This structure appears when the fungus is searching for nutritive resources. On this medium, spore production is not possible since their formation and subsequent germination require, in most filamentous fungi, the availability of nutrients in the culture medium, such as sugars, amino acids and inorganic salts [40].

On BOA (+), the color of the mycelium becomes black resulting from sporulation (Figure 1b,c). When exposed to wavelengths between 355 and 495 nm, followed by a dark period on rich medium, the fungus produces a great number of spores [41,42]. Spores of *D. teres* are cylindrical in shape with round ends, having a length from 25 to 300 µm and thickness from 7–11 µm [4,5]. Due to their septa, these spores are recognizable from other fungi (Figure 1d) [43]. The spores presenting less than two septa will not germinate and cannot penetrate plant tissues [44]. *D. teres* infects the plants via the spores, the reproductive structures, which are dispersed largely by the wind or rain and often over long distances.

On PDA (+/−), *D. teres* covers the entire surface of the culture medium within seven days, denoting a rapid growth (Figure 1e). On this medium, *D. teres* produces a very small number of spores (Figure 1g) as compared to the BOA (+) medium (Figure 1b) [45]. Since the PDA (+/−) medium is suitable for the development of bacteria and fungi, a co-culture of *D. teres* with PsJN, was performed. When grown alone on the PDA (+/−) medium (Figure 1e), *D. teres* has a fluffy mycelium with some feather dusters characteristic of this fungus [46]. Under co-cultivation with PsJN, *D. teres* has a different phenotype than when growing alone on PDA (+/−) (Figure 1f). Indeed, the mycelium of *D. teres* is less fluffy and fruiting bodies are present at the periphery, probably to provide a protective barrier against PsJN. PsJN protects indeed several crops against damages caused by different abiotic or biotic stresses and promotes plant growth [17,18,19,47,48,49,50]. According to our results, this strain seems to have no antifungal effect and is, therefore, unable to prevent the development of *D. teres*.

The results suggest that the variability of the phenotypes observed on the different media would be accompanied by changes in the expression of cell-wall-related genes, since the fungal cell wall is a dynamic structure accommodating the different growth stages and morphologies.

### 3.2. Identification of CHS Genes in D. teres and Phylogenetic Analysis

The fungal cell wall is a complex and dynamic structure that protects the cell from environmental stresses [10,51]. Given the important role played in fungal physiology, the cell wall is considered as a suitable target for antifungal drugs [6].

Chitin is the most important structural component of the fungal cell wall [9,10,11,52,53]. The enzymes catalyzing the synthesis of chitin are CHSs, which are members of glycosyltransferases from family 2 (GT2), like cellulose synthases. CHSs are able to transfer *N*-acetyl-D-glucosamine from an activated sugar donor (UDP-*N*-acetyl-D-glucosamine) to an elongating chitin chain [6,54].

In previous work, seven chitin synthase genes *chsA*, *chsB*, *chsC, chsD*, *chsE*, *csmA* and *csmB* were identified in the model organism *A. nidulans* encoding CHSs of Classes I, II, III, IV, V VI and VII [22]. The presence of multiple CHSs in many fungi suggests that several *CHS*s can be used for chitin production at different stages of the fungal life-cycle [10].

According to the classification proposed by Chigira et al. [55] and Choquer et al. [56], in our study, *DtCHS1*, *DtCHS2*, *DtCHS3*, *DtCHS4*, *DtCHS5* and *DtCHS7* encode CHSs from Class I, Class II, Class III, Class IV, Class V and Class VII, respectively [55,56]. These enzymes are localized in the plasma membrane [9].

**Figure 2:**
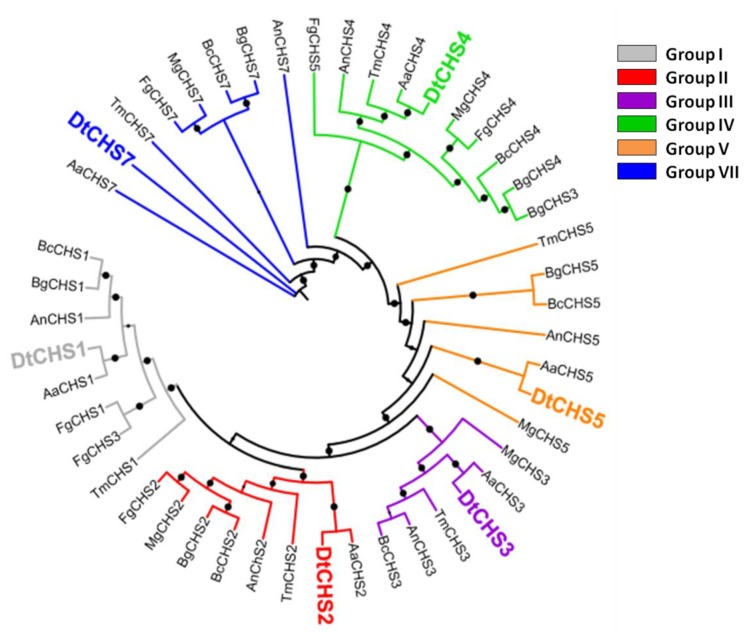
Maximum likelihood phylogenetic tree (bootstraps: 100) of chitin synthases (CHS) from *D. teres* (*Dt*), *A. nidulans* (*An*), *A. alternata* (*Aa*), *B. cinerea* (*Bc*), *B. graminis* (*Bg*), *F. graminearum* (*Fg*), *T. melanosporum (Tm*) and *M. grisea* (*Mg*). The protein accession numbers are indicated in Table 2. Bootstraps > 80% are represented as black circles on the branches. The bootstrap % value increases with the circle size.

BLASTp analysis carried out using the CHS protein sequences of *A. nidulans* identified six CHSs in *D. teres* (hereafter referred to as DtCHS1, 2, 3, 4, 5 and 7 for the protein sequences and *DtCHS1, 2, 3, 4, 5* and *7* for the genes) (Table 1). The maximum likelihood phylogenetic analysis carried out using CHS full-length protein sequences from several classes of fungi, notably *Dothideomycetes*, *Sordariomycetes* and *Leotiomycetes*, allowed assigning a phylogenetic relatedness for the *D. teres* CHS with known orthologs from other species (Figure 2). 

The phylogenetic analysis demonstrates the existence of six CHS classes (Figure 2). Class I, II and IV are present in all fungi, while Classes III, V and VII are particular to filamentous fungi [57]. The number of *CHS* genes changes according to the species. Most fungal species contain between three and six CHS genes [58]. For example, three *CHS* are present in *S. cerevisiae*, four in *Candida albicans* and eight in *A. nidulans* [59,60].

CHS1 and CHS2 have overlapping functions in septum formation in *A. nidulans* [61]. In the same way, CHS1 is crucial for infection-related morphogenesis, since 90% of the *chs*1 *M. oryzae* mutants have no septum and, therefore, display severe defects in conidium morphology [9]. CHS1 is also essential for cell wall integrity in *Candida albicans* [59]. Class IV enzymes contribute to the synthesis of the bulk cell wall chitin [57]. In *M. oryzae*, CHS1 is important for virulence and plays specific roles during conidiogenesis and appressorium formation. CHS2, CHS3, CHS4 and CHS5 are essential for plant infection and CHS6 is dispensable for pathogenesis [9]. Therefore, individual *CHS* genes play several roles in hyphal growth, pathogenesis, conidiogenesis and appressorium development.

**Figure 3:**
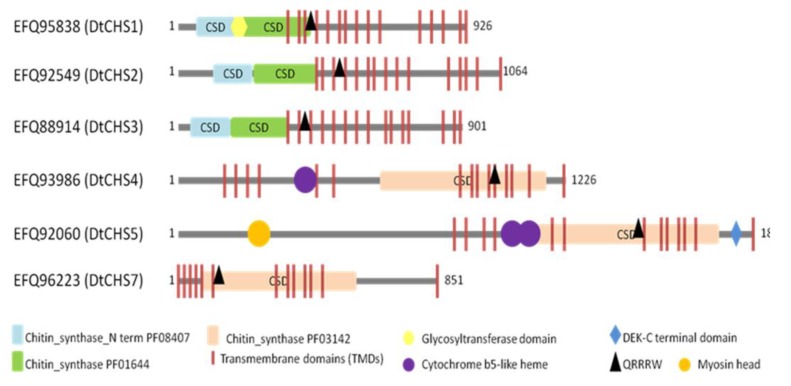
Schematic representation showing the domain organization of the six putative CHSs from *D. teres*. Highlighted motifs were identified with Motifscanner and predicted transmembrane domains were identified with Phobius and TMHMM programs. CSD: Chitin Synthase Domain.

**Figure 4:**
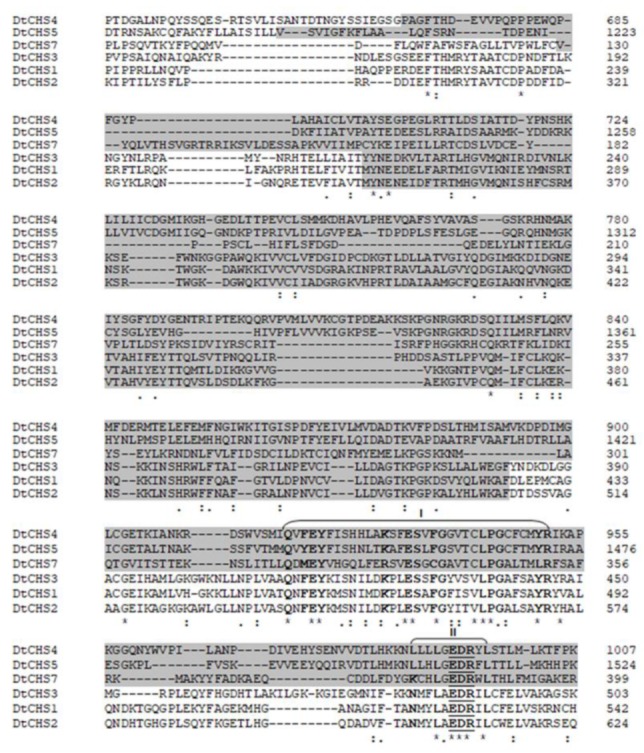

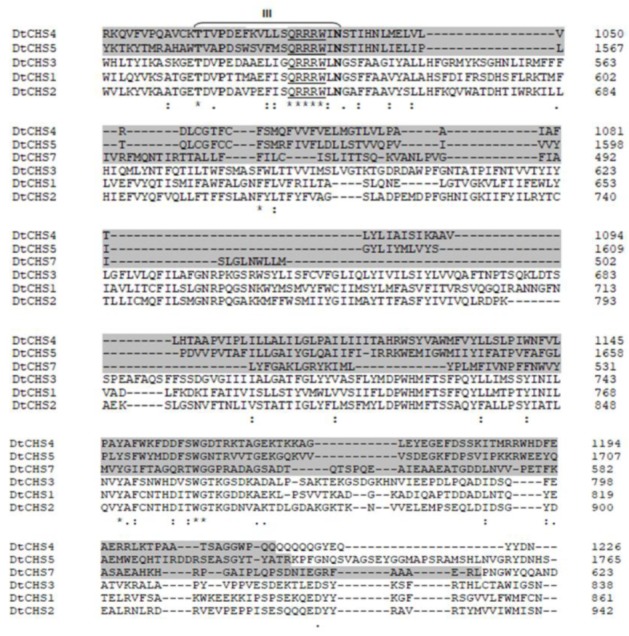
Partial amino acid sequence alignment of the six *D. teres* CHSs showing the conserved chitin synthase domain (highlighted) and “QRRRW”/“EDR” motifs (underlined). Amino acids indicated with bold characters are those conserved in all CHSs listed here. I-III represent subdomains where conserved amino acids appear at high frequencies.

BLASTp analyses and motif searches reveal similarities between the six identified CHSs from *D. teres* and other fungal CHSs (Table 3 and Figure 3 and Figure 4). Structurally, CHS1, CHS2 and CHS3 are more similar to each other than to other CHSs in *D. teres*. As in *A. nidulans* and other filamentous fungi, Class V CHSs in *D. teres* have myosin motor-like domains at the N-terminus [11,62]. Myosins are actin-dependent molecular motors and play roles in several cellular processes. More specifically, the head myosin domain binds to actin in an ATP-dependent manner and generates force by ATP hydrolysis [63].

CHSs have several conserved domains which are considered as signature sequences since they are found in all CHSs [64]. Most of the amino acids of these signature motifs were found to be essential for activity [65]. CHSs contain the conserved EDR motif and the pentapeptide QRRRW (Figure 4) which was reported also in CHSs from the chordate *Branchiostoma floridae* [66]. The importance of these motifs has been studied in yeast. Thereby, in *Saccharomyces cerevisiae*, mutations of the QRRRW motif lead to a significant decrease in CHSs activity [58]. By comparing the amino acid sequences of the six *D. teres* CHSs in NCBI, DtCHS1/DtCHS2, DtCHS2/DtCHS3 and DtCHS4/DtCHS5 have similarities, with 43%, 44% and 45% identity, respectively (Figure 3 and Figure 4). DtCHS4 and DtCHS5 are closer with respect to the similarity of amino acid sequences. This sequence similarity is also confirmed by the phylogenetic tree (Figure 2).

### 3.3. Expression Analysis of Cell Wall-Related Genes in D. teres

The software geNorm^PLUS^ [67] was used to analyze gene expression stability across different samples of *D. teres*: spores and mycelium on BOA (+), mycelium on MP (−), and mycelium on PDA (+/−) in the presence or absence of the bacterium. The stability and transcript levels of the eleven candidate reference genes *actin*, *ApsC, Cos4, GlkA, PfkA, PgiA, SarA, IsdA, H2B, GAPDH* and *RS14* were investigated with geNorm^PLUS^. *Cos4* and *PgiA* were identified as the best two transcripts for use in normalization of the data (Supplementary Figure S1).

The attention was focused on key genes involved in fungal cell wall biosynthesis. Besides *CHS*s, genes coding for enzymes involved in β-(1,3;1,4)-glucan and β-(1,3)-glucan synthesis were investigated. Indeed, β-(1,3)-glucan is relevant since it accounts for 65–90% of the total β-glucan content [68].

The β-(1,3)-glucan hydrolyzing enzymes can be separated into exo-β-(1,3)-glucanases and endo-β-(1,3)-glucanases according to their activities [68]. Endo-β-(1,3)-glucanases cut within the chain of glucans, whereas exo-β-(1,3)-glucanases cleave the residues of glucose at the end of the chain [69,70]. During fungal growth, β-(1,3)-glucanases are essential for the remodeling of the cell wall [69]. β-(1,3)-glucans are synthesized by a glucan synthase complex, which uses UDP-glucose as a substrate and extrudes β-(1,3)-glucan chains [70]. *FksA* encodes the catalytic subunits of the glucan synthase complex and is cell-cycle-regulated [6,71].

The cell wall of ascomycetes also contains β-(1,3;1,4)-glucan accounting for 10% of the glucan content in the *A. nidulans* cell wall [35,72]. *celA* encodes a putative mixed-linkage glucan synthase in *A. nidulans* [22]; one ortholog was found in *D. teres* and was used in this study.

We also considered a gene encoding a protein kinase, *PTK1*, which was shown to be required for conidiation, appressoria formation and pathogenicity in *D. teres* [36].

**Figure 5:**
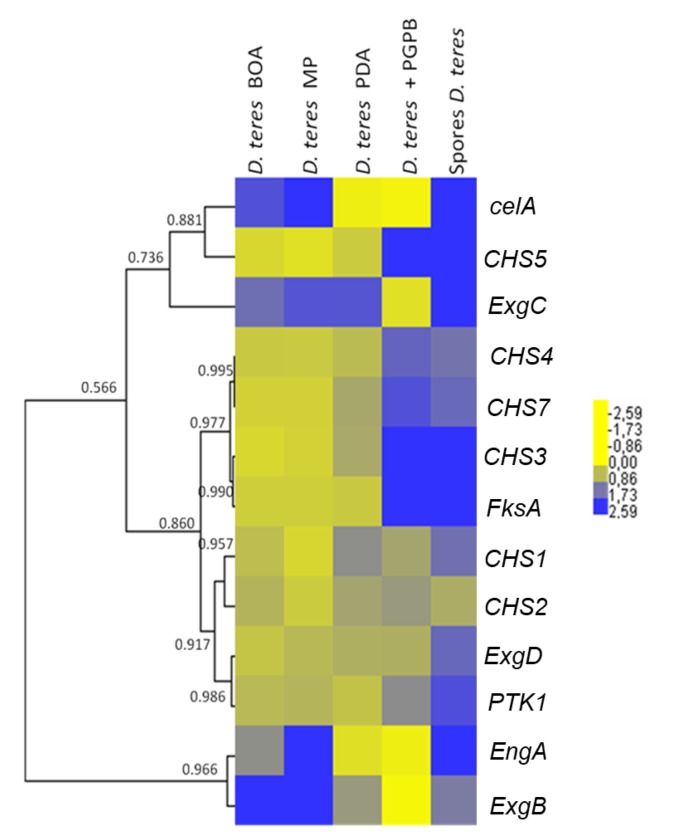
Heatmap hierarchical clustering with correlation coefficients of genes related to cell wall synthesis in *D. teres*. The heat map hierarchical clustering was generated using Pearson correlation in complete linkage.

The hierarchical clustering of the heat map (Figure 5) shows that the *CHS* genes studied group in different clusters. *CHS3*, *CHS4*, *CHS5* and *CHS7* show a higher expression in spores and in the mycelium grown in the presence of the bacterium. This suggests that the bacterium induces the expression of specific *CHS*s in the phytopathogenic fungus. This phenomenon could be explained by an effect on the cell wall of the fungus. Indeed, PGPB is able to produce different types of cell-wall-lysing enzymes including chitinases, proteases, cellulases and β-(1,3)-glucanases [73,74]. The fungus could respond to PsJN by increasing the expression of *CHS*s in the attempt to restore the structural integrity of its cell wall.

**Figure 6:**
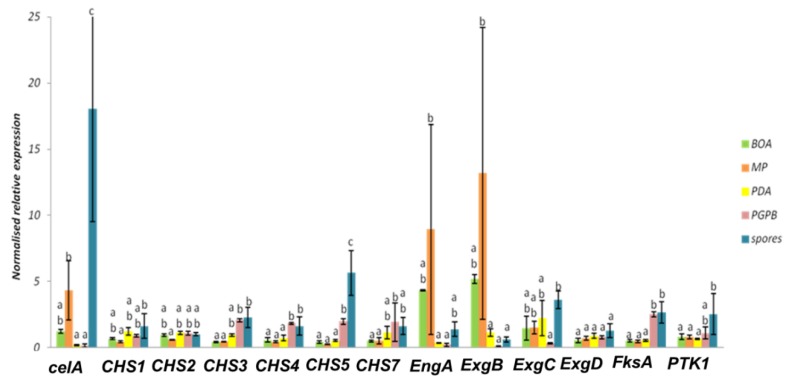
Normalized relative expression of cell wall genes of *D. teres* cultivated on different media. Different letters indicate statistically different values (*p*-value < 0.05) among the groups of the one-way ANOVA with Tukey’s post-hoc test. Error bars represent the standard deviation of four independent biological replicates.

Some *CHS* genes, such as *CHS1* and *CHS2*, showed a tendency towards decreased expression on the poor medium MP (-) (Figure 5 and Figure 6). This finding may indicate that these genes have a role in vegetative growth and the lower expression reflects the slower mycelium growth rate in a nutrient-poor environment.

In *D. teres*, *EngA* is more expressed in the mycelium cultivated on the poor medium (MP (-) medium) (Figure 5 and Figure 6). Likewise, the exo-β-(1,3)-glucanases *ExgB* is expressed at higher levels on the poor medium (MP (-) medium) (Figure 5 and Figure 6). However, the differences are not statistically significant and only show a trend. 

*FksA* is more expressed in spores and the mycelium with PsJN (Figure 5 and Figure 6). According to Figure 6, the expression of *celA* is significantly higher in *D. teres* spores, as compared to the other conditions.

*PTK1* has a slightly larger expression in the spores as compared to the other experimental conditions (Figure 5 and Figure 6). This is in agreement with the reported role of *PTK1* in conidiation [36].

Our results show that the expression of *CHS4*, *CHS5* and *FksA* is higher in the mycelium cultivated on PDA (+/−) medium with PsJN, as compared to the growth on BOA (+), MP (−) and PDA (+/−) (Figure 6). According to Figure 1, PsJN has an effect on the phenotype of *D. teres*. This could be due to a possible secretion of hydrolytic enzymes by PsJN, acting on the integrity of the fungal cell wall [73,74]. *D. teres* could also perceive PsJN as a stress and thus strengthen its cell wall. 

This hypothesis will have to be confirmed and validated by future experiments.

## 4. Conclusions

To the best of our knowledge, this is the first study devoted to the cell wall-related genes of *D. teres*. We identify key genes involved in the biosynthesis/remodeling of *D. teres* cell wall and differentially expressed in spores and/or in the mycelium depending on the culture media used. We also identify some cell wall biosynthetic genes induced by PsJN, a plant growth-promoting bacterium. Since PsJN seems to disturb fungal growth, it is reasonable to hypothesize that it could produce cell wall degrading enzymes causing a response in *D. teres* at the gene level. Additionally, we propose a list of potential candidate reference genes for qPCR analysis in *D. teres*. Our study paves the way to follow-up studies aiming at a functional characterization of cell wall genes of this economically relevant pathogen.

## Figures and Tables

**Table 1:** List of the chitin synthase (CHS) protein accession numbers from the species used in this study.

Species of Fungi	Protein Id	Accession Number
*Drechslera teres*	DtCHS1	EFQ95838
	DtCHS2	EFQ92549
	DtCHS3	EFQ88914
	DtCHS4	EFQ93986
	DtCHS5	EFQ92060
	DtCHS7	EFQ96223
*Aspergillus nidulans*	AnCHS1	P30583
	AnCHS2	P30584
	AnCHS3	XP_660127
	AnCHS4	P78611
	AnCHS5	XP_663922
	AnCHS7	XP_658650
*Alternaria alternata*	AaCHS1	XP_018390769
	AaCHS2	XP_018389428
	AaCHS3	XP_018384374
	AaCHS4	XP_018387091
	AaCHS5	XP_018384599
	AaCHS7	XP_018384594
*Botrytis cinerea*	BcCHS1	XP_024550705
	BcCHS2	XP_001550325
	BcCHS3	XP_001557191
	BcCHS4	XP_024546183
	BcCHS5	XP_001545514
	BcCHS7	XP_024549635
*Blumeria graminis*	BgCHS1	EPQ66343
	BgCHS2	EPQ67341
	BgCHS3	EPQ67743
	BgCHS4	CCU76828
	BgCHS5	AAF04279
	BgCHS7	CCU74227
*Fusarium graminearum*	FgCHS1	CAC41025
	FgCHS2	XP_011318411
	FgCHS3	PCD18709
	FgCHS4	XP_011317052
	FgCHS5	XP_011317820
	FgCHS7	XP_011317804
*Tuber melanosporum*	TmCHS1	XP_002842229
	TmCHS2	XP_002840530
	TmCHS3	XP_002837735
	TmCHS4	XP_002840095
	TmCHS5	XP_002839897
	TmCHS7	XP_002835817
*Magnaporthe grisea*	MgCHS2	CAA65275
	MgCHS3	CAA65276
	MgCHS4	AAB71411
	MgCHS5	BAA74449
	MgCHS7	ACH58563

**Table 2:** Description of the primers used for the qPCR analyses with details on the sequences, amplicons’ length, Tm, amplification efficiency and regression coefficient.

Name	Sequence (5’–3’)	Amplicon Length (bp)	Amplicon Tm (°C)	PCR Efficiency (%)	Regression Coefficient (R^2^)
Actin Fwd	ATGTTGGTGATGAGGCACAG	123	84.4	87.5	0.999
Actin Rev	GCTCGTTGTAGAAGGTGTGATG				
ApsC Fwd	TCACCGATTCAGGTCTCAAC	148	85.8	87.8	0.998
ApsC Rev	ATGTTGGGGCTCTTGATGTC				
Cos4 Fwd	GCACACTTCTCCCCAGAGC	104	87.8	89.6	0.999
Cos4 Rev	CCATCGCTTCTCGATATTGG				
GAPDH Fwd	AGGGCAAACTGAACGGTATC	92	82.9	93.9	0.998
GAPDH Rev	GGCATCGAAAATGGAAGAGC				
GlkA Fwd	CGCTTGGAACTGCTTTCTTC	106	86.8	93.8	0.995
GlkA Rev	TGTAGGACGATTGGGTTTCG				
PfkA Fwd	GTTCCCAGCCCAGTTATTTG	93	81.9	91.3	0.998
PfkA Rev	AGCAACAGCGACTTCTTTGG				
PgiA Fwd	CAACTTCCACCAACTTCTCG	98	82.9	95.0	0.999
PgiA Rev	TTAGCAGACCACCAATGACG				
SarA Fwd	AGATGCCATTTCCGAGGAC	116	86.4	92.4	0.997
SarA Rev	CCACACTGCACATGAAGACC				
IsdA Fwd	TCAAGAAGATGTGGCTGTCG	89	85.3	86.0	0.999
IsdA Rev	GATGGTGGGGATGACAATG				
H2B Fwd	TACAAGGTCCTCAAGCAGGTC	96	83.8	90.6	0.997
H2B Rev	AACACGCTCGAAGATGTCG				
RS14 Fwd	CACATCACCGATCTTTCTGG	146	88.2	87.0	0.997
RS14 Rev	GTAATGCCGAGTTCCTTGC				
PTK1 Fwd	TGCTCCTAAACGCAAACTGC	93	84.2	92.0	0.999
PTK1 Rev	CCGTCATGAATCCAGAGTTG				
CHS1 Fwd	GGACATCAAAAAGGGTGTCG	119	82.2	89.4	0.999
CHS1 Rev	ATGCCTGGAAGAACCATCTG				
CHS2 Fwd	TCCAAGAGGGTATTGCGAAG	102	82.3	102.0	0.988
CHS2 Rev	TGAATTTGAGGTCCGAGTCC				
CHS3 Fwd	GCCTGAAGCAAAAGAACAGC	90	83.2	91.4	0.999
CHS3 Rev	AAATGCAGACTTCGGGGTTC				
CHS4 Fwd	TCATCATCTGCGACGGTATG	115	83.3	94.0	0.996
CHS4 Rev	GAAAATGCCTGAACCTCGTG				
CHS5 Fwd	CAAGTGCGTTCGTCAACAAG	107	83.4	93.0	0.999
CHS5 Rev	GTCCAAGAAACTCGGCAAAC				
CHS7 Fwd	CGGAAAAGAACTCGCTCATC	141	85.8	91.2	0.998
CHS7 Rev	GGAAAGCAGAGAATCGCAAC				
FksA Fwd	AGTTTCTTACGCTGGCAACC	146	89.5	89.0	0.999
FksA Rev	CTTCCTTGGTACAGGGAATCTG				
EngA Fwd	TCAAGTGTGGAAGGGTATCG	107	84.8	93.3	0.994
EngA Rev	AGCCGTAATGGAAGTGATGG				
ExgB Fwd	TGGATGATGGGAGATGAGTG	90	82.0	102.8	0.996
ExgB Rev	GGCCTTTGTTTGACCAAGTG				
ExgC Fwd	TAAACACAGGCGGATGGTTC	145	88.1	91.3	0.994
ExgC Rev	CACGAATTCCAGTGGTCTTG				
ExgD Fwd	GACGCAAACGAAGAGAATCC	101	86.3	88.2	0.998
ExgD Rev	TGGTAGTAAATCGCCCTGTG				
celA Fwd	CATTACCGCCCTTTTGTCAC	117	81.0	88.7	0.998
celA Rev	ATGAAGAACCACGCAAGACC				

**Table 3:** *D. teres* CHS with predicted transmembrane domains (TMDs), deduced protein lengths and predicted domains.

CHS	No. of TMDs	Length	Domains (*E*-values)
CHS1	15	926	Glycosyltransferase (0.0011), Chitin synthase (1,7e^−152^), Chitin synthase N-terminal (1,5e^−53^)
CHS2	15	1064	Chitin synthase (3,3e^−153^), Chitin synthase N-terminal (3,1e^−43^)
CHS3	15	901	Chitin synthase (4,6e^−123^), Chitin synthase N-terminal (4e^−53^)
CHS4	15	1226	Cytochrome b5-like heme (6.8e^−10^), Chitin synthase (0)
CHS5	13	1844	Cytochrome b5-like heme (2.8e^−15^), Chitin synthase (0), DEK-C terminal domain term (5.3e^−24^), Myosin-head (3.9e^−165^)
CHS7	13	851	Chitin synthase (9.9e^−7^)

## Supplementary Materials

The following is available online at https://www.mdpi.com/2073-4425/11/3/300/s1, Figure S1: Ranking of eleven candidate reference genes in *D. teres* according to the parameter M computed by geNorm^PLUS^. The increase in stability of the candidate genes is determined by a decrease in the M value.
