# Supplementary material for: Expression Analysis of Cell Wall-Related Genes in the Plant Pathogenic Fungus Drechslera teres
Source: Genes (Basel). 2020 Mar 12;11(3):300. doi: 10.3390/genes11030300 (PMC7140844; doi:10.3390/genes11030300)
Supplement: Supplementary file 1 [file genes-11-00300-s001.pdf]

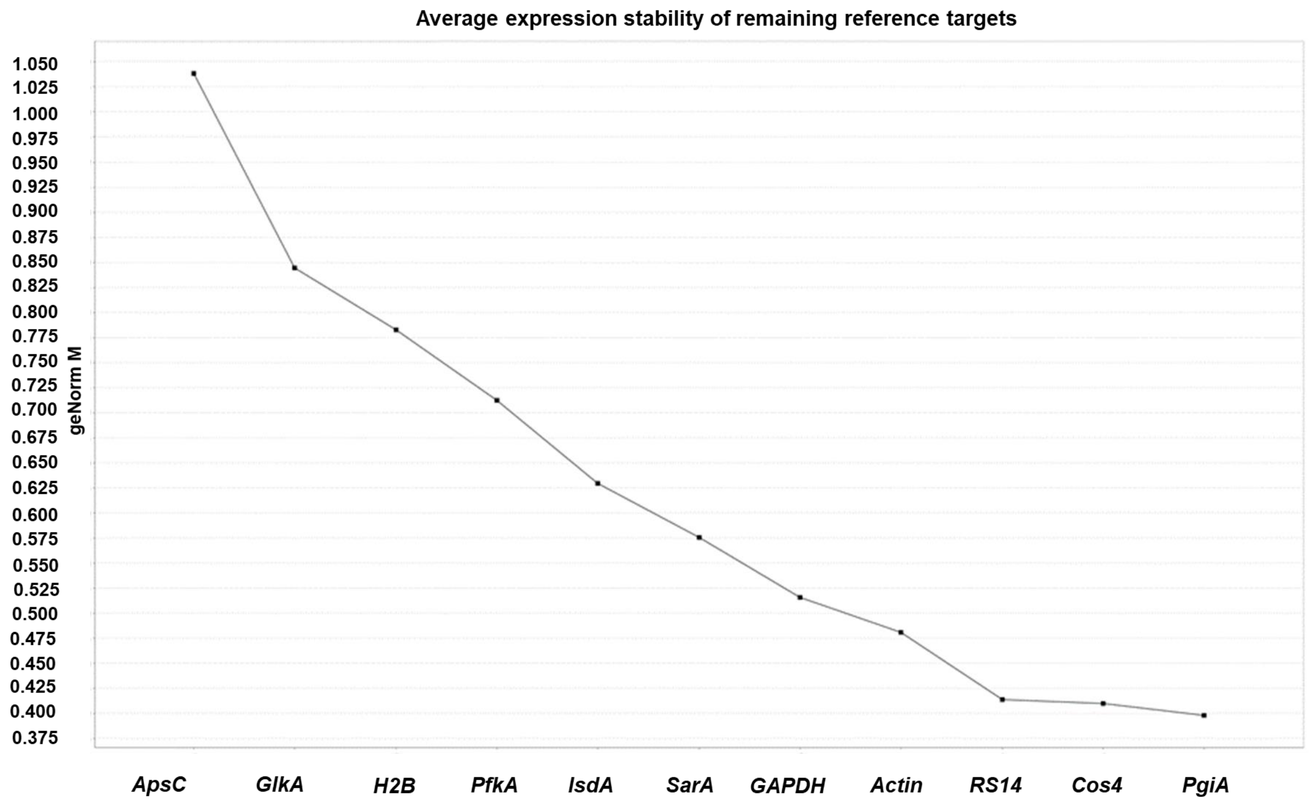

Supplementary Figure 1: Ranking of eleven candidate reference genes in *D. teres* according to the parameter M computed by geNorm^{PLUS}. The increase in stability of the candidate genes is determined by a decrease in the M value.
